# Epidemic Small Pox

**Published:** 1846-02

**Authors:** 


The following extract from the Medical Examiner of January contains some very judicious remarks upon vaccination, and is justly severe upon a large class of our fellow citizens for their "obstinacy," in refusing, by their negligence, to appreciate its importance.

*Epidemic Small-Pox.*—We have never known small-pox to be so prevalent throughout the country as at the present time. Cities, towns, and villages, every where, are infested with it to a great extent; and what is remarkable, the epidemic seems to be as mild as it is prevalent. The great majority of cases occur in persons who have undergone a degree of protection by having previously had the disease or been vaccinated, and in such, as usual, it is greatly modified—the attack consisting of more or less pain in the head and back, some nausea, fever for a day or two at the commencement, with a sparse eruption, and *no secondary fever.* Such cases require very little treatment, recover in from three to five or six days after the first appearance of the eruption, and are followed by no disfiguration. When the disease attacks those who have not previously been vaccinated successfully, or have not had the variolous disease, it runs the ordinary course of unmitigated small-pox; in some instances being discrete, and in others confluent, according to the constitution and treatment cf the patient. In Philadelphia, where the disease has been quite prevalent for more than a month past, we have heard of no instance in which it has proved fatal where the subject was known to have been successfully vaccinated, and the deaths that have occurred, as far as have come to our knowledge, have been confined to such as had never been vaccinated, or in whom the proper vaccine mark had disappeared, if it had ever existed. It is a subject of astonishment and regret, that in an enlightened community like that in which we live, so much laxity and obstinacy should prevail in regard to the necessity of vaccination. In repeated instances, since the present epidemic has appeared, we have had occasion to vaccinate two and three persons in one family, mostly children or servants, who had until the time been neglected. How can it be expected that we shall be exempt for any long time from a disease so communicable, while such carelessness and stupidity prevails? A large proportion of the unprotected cases that occur in Philadelphia, are in persons who have come hither from remote or surrounding places, and it would appear that an equal degree of the carelessness to which we have referred, prevails all over the country. Even in the Eastern States, among a people so proverbial for their prudence, the same heedlessness prevails.

